# Transient receptor potential canonical 4 and 5 proteins as targets in cancer therapeutics

**DOI:** 10.1007/s00249-016-1142-1

**Published:** 2016-06-11

**Authors:** Hannah J. Gaunt, Naveen S. Vasudev, David J. Beech

**Affiliations:** School of Medicine, University of Leeds, LIGHT Building, Clarendon Way, Leeds, LS2 9JT UK

**Keywords:** Ion channel, Calcium ion, Non-selective cationic channel, Transient receptor potential canonical, Cancer cell, Renal cell carcinoma, Breast cancer, Ewing’s sarcoma, Englerin A

## Abstract

Novel approaches towards cancer therapy are urgently needed. One approach might be to target ion channels mediating Ca^2+^ entry because of the critical roles played by Ca^2+^ in many cell types, including cancer cells. There are several types of these ion channels, but here we address those formed by assembly of transient receptor potential canonical (TRPC) proteins, particularly those which involve two closely related members of the family: TRPC4 and TRPC5. We focus on these proteins because recent studies point to roles in important aspects of cancer: drug resistance, transmission of drug resistance through extracellular vesicles, tumour vascularisation, and evoked cancer cell death by the TRPC4/5 channel activator (−)-englerin A. We conclude that further research is both justified and necessary before these proteins can be considered as strong targets for anti-cancer cell drug discovery programmes. It is nevertheless already apparent that inhibitors of the channels would be unlikely to cause significant adverse effects, but, rather, have other effects which may be beneficial in the context of cancer and chemotherapy, potentially including suppression of innate fear, visceral pain and pathological cardiac remodelling.

## Introduction

Despite advancements in prevention strategies, diagnostics and therapeutics, cancer remains a major worldwide health problem. Unacceptably high rates of treatment failure exist, often due to the adaptable nature of tumour cells. In many cases, localised non-metastatic cancers can be treated with surgery alone, but for those that relapse or present with metastatic disease, systemic treatment options are typically required. Chemotherapy is a common approach, but this is associated with only modest benefit in most solid tumours and it is ineffective in others, such as renal cell carcinoma. More recently, a number of small-molecule kinase inhibitors have been introduced, but these agents are invariably associated with either innate or acquired drug resistance. Furthermore, currently used therapies are frequently associated with significant adverse effects. Novel pharmacological targets and approaches for cancer therapy are in high demand, and various ion channels have been suggested as potentially useful targets for therapies; notable amongst them are the channels which are permeable to Ca^2+^ and which therefore often allow Ca^2+^ entry into cells (Arcangeli et al. 2009; Monteith et al. 2012; Prevarskaya et al. 2011).

There are many types of Ca^2+^-permeable channel, but here we focus on those formed by assembly of transient receptor potential canonical (TRPC) proteins and particularly those which involve two closely related members of the family: TRPC4 and TRPC5. We focus on these proteins because recent studies suggest that they have roles in several important aspects of cancer: drug resistance, transmission of drug resistance through extracellular vesicles, tumour vascularisation, and evoked cancer cell death. We describe the experimental evidence and discuss the implications for future potential studies and therapeutic strategies. We address not only the potential direct relevance to cancer cells but also relevance to other aspects of biology which are important for many cancer patients.

### Control of cell function by intracellular Ca^2+^

This review focusses on the Ca^2+^ permeability of TRPC channels. Ca^2+^ enters cells through a variety of ion channels and is particularly recognised for its importance as a versatile dynamic intracellular regulator of mammalian cell biology (Berridge et al. 2000; Clapham 2007). Finely tuned control of the free intracellular Ca^2+^ concentration is fundamental for the survival and death of cells in mammals as well as many other types of animal. Plasma membrane ion channels that are permeable to Ca^2+^ and allow Ca^2+^ entry down its steep gradient often have positive, although not necessarily beneficial, effects on cells which include increased proliferation, migration and invasiveness (Clapham 2007; Chen et al. 2013). Excessive elevation of intracellular Ca^2+^ is conversely associated with cytotoxicity (Clapham 2007; Berridge et al. 2000; Fleckenstein et al. 1974; Orrenius et al. 2003).

### Transient receptor potential (TRP) proteins and channels

TRPs are membrane proteins which assemble as tetramers around a central ion pore to form non-selective cationic channels, many of which are Ca^2+^ permeable. There are 28 genes encoding the different TRPs. A greater number of channels exist because of heteromeric assembly involving more than one type of TRP. Although there is differential expression of TRPs in different cell types and tissues, most TRPs are quite broadly expressed. They are expressed in excitable cells, where they contribute positively to electrical excitability alongside voltage-gated Ca^2+^ channels, and in non-excitable cells, where they promote migration and proliferation and other relatively slow cell changes (Zheng and Phelan 2014; Abramowitz and Birnbaumer 2009; Al-Shawaf et al. 2010; Zeng et al. 2013). The initial discovery of TRP channels arose in photo-transduction studies in *Drosophila melanogaster*. Mutation in this fly’s TRP gene resulted in a transient, rather than the normally sustained, membrane depolarisation in response to bright light, hence the name transient receptor potential (Minke et al. 1975). Many mammalian homologues were subsequently discovered and cloned, starting with TRP canonical 1 (TRPC1) (Wes et al. 1995; Birnbaumer 2009; Beech 2013; Bon and Beech 2013). The TRP super-family is now categorised into subgroups: TRP canonical (TRPC), TRP vanilloid (TRPV), TRP melastatin (TRPM), TRP ankyrin (TRPA), TRP polycystin (TRPP) and TRP mucolipin (TRPML) (Damann et al. 2008; Birnbaumer 2009).

Voltage-gated channels are considered the proteins most structurally related to the TRPs; akin to the K_V_1.2 voltage-gated potassium channel, for which there is a crystal structure, all TRPs are suggested to have six membrane-spanning segments and intracellular N- and C-termini. Like K_V_1 channels, TRP channels may arise from either four identical or four different members of the family (i.e. they may be homotetramers or heterotetramers). Experimental tests of this hypothesis have supported this topology, and cryo-electron microscopy (EM) structural data for two of the TRPs, TRPV1 and TRPA1, have further corroborated it (Liao et al. 2013; Paulsen et al. 2015).

### Channels which contain TRPC4 and TRPC5

There are seven TRPC types in mammals, and all are considered to contribute to plasma membrane non-selective cationic channels which confer Na^+^ as well as Ca^2+^ permeability. The Na^+^ entry may contribute functionally by helping to depolarise the membrane potential and elevate intracellular Ca^2+^ indirectly via Na^+^–Ca^2+^ exchange. In humans and the great apes, TRPC2 protein is absent, being encoded by a pseudogene in these species (Vannier et al. 1999; Damann et al. 2008; Abramowitz and Birnbaumer 2009). The TRPCs are notable amongst the TRPs for being likely to exist as heteromers. TRPC1 may not form functional homomeric channels at all, yet there is compelling evidence for its distinctive and important contributions to heteromers with TRPC4 and TRPC5. Although TRPC4 and TRPC5 are capable of forming homomeric channels, TRPC1 is very broadly expressed, and so they probably commonly exist physiologically as heteromers with TRPC1. Further promiscuity has been suggested, even outside the TRPC family, stretching to TRPV4 and TRPP2 (Bai et al. 2008; Ma et al. 2010; Strubing et al. 2001; Sukumar et al. 2012; Xu et al. 2008).

The TRPC channels almost certainly do not have a single physiological activator. As with other TRP channels, there is promiscuity, also called versatility, of activation, which means that multiple activators have been identified and several are often relevant in physiology and patho-physiology, suggesting context-dependent activation. Also consistent with concepts for other TRPs, there are examples where modulators of TRPC channels are not endogenous physiological factors but exogenous chemicals from plants, suggesting that the channels act at least in part to integrate humans and other mammals with the external environment. Modulators of the channels include receptor agonists, hydrogen peroxide, mild acidification, toxic metal ions, oxidised phospholipids, galangin and ω-3 fatty acids (Beech 2013; Tominaga et al. 1998; McKemy 2005; Jordt et al. 2004; Akbulut et al. 2015; Sukumar et al. 2012; Naylor et al. 2015).

### Tools for studying roles of TRPC4- and TRPC5-containing channels

Selective and potent pharmacological agents to modulate TRPC4 and TRPC5 channels have been lacking, but this is an active area of investigation and so better tools should be available in the near future for exploring the roles of the channels (Bon and Beech 2013). Some commonly used small-molecule inhibitors such as SKF-93635 and 2-APB are non-specific, and the arising data not of great value. ML204 and clemizole hydrochloride are more recently identified and more specific, inhibiting the channels in the micromolar concentration range (Miller et al. 2011; Richter et al. 2014a). Small-molecule activators of the channels include riluzole and rosiglitazone, but again these are non-specific and lack potency (Jung et al. 2003; Majeed et al. 2011; Richter et al. 2014b; Flemming et al. 2006). Recently we discovered the natural product (−)-englerin A as the first selective and potent small-molecule activator of TRPC4- and TRPC5-containing channels (Akbulut et al. 2015). High-quality small-molecule modulators are especially valuable for understanding the roles of the channels in human tissues and cells obtained from clinical samples.

Short interfering and short hairpin RNAs are used for studying the channels in cells which can be transfected or which are suitable for viral delivery methods (Carson et al. 2015; Ma et al. 2014; Stewart et al. 2015). There are mice available with disrupted TRPC4 or TRPC5 genes (Phelan et al. 2013; Tsvilovskyy et al. 2009). Extracellularly acting inhibitor antibodies have been developed to TRPC1, TRPC4 and TRPC5, which have been useful in revealing roles of the channels (Sukumar et al. 2012; Xu et al. 2005, 2006; Mohl et al. 2011; Akbulut et al. 2015).

### Cancer-independent roles of TRPC4- and TRPC5-containing channels

Despite the limitations of the TRPC4 and TRPC5 tools, there is compelling evidence for important roles of TRPC4- and TRPC5-containing channels, especially in animal models of human patho-physiology or in clinical samples. The channels have positive roles in epilepsy, innate fear, pain, adverse cardiac remodelling as well as other aspects of physiology and patho-physiology (Zheng and Phelan 2014; Phelan et al. 2013; Riccio et al. 2014; Westlund et al. 2014; Bon and Beech 2013; Wei et al. 2015; Camacho Londono et al. 2015). TRPC5 knockout mice exhibited reduced innate fear (Riccio et al. 2014), and TRPC4 knockout mice presented with diminished anxiety (Riccio et al. 2014). TRPC1 was up-regulated and had a positive role in neointimal hyperplasia of human saphenous vein, where it may function in partnership with TRPC5 (Kumar et al. 2006; Xu et al. 2006). In vascular smooth muscle cells from human saphenous vein, TRPC5-dependent channels were activated by sphingosine-1-phosphate and helped to drive cell migration (Xu et al. 2006). TRPC5 was also implicated in kidney barrier function, protecting against albuminuria (Schaldecker et al. 2013). TRPC4 was required for the transmission and detection of the colonic visceral pain sensation associated with application of mustard oil. TRPC4^−/−^ mice and mice treated with the TRPC4 inhibitor ML204 showed less lower-body licking and abdominal retractions in response to application of mustard oil (Westlund et al. 2014). Amygdaloid TRPC4 and TRPC5 contributed to maintenance of pain hypersensitivity and neuropathy (Wei et al. 2015).

## Direct relevance of TRPC4 and TRPC5 to cancers?

Malignant transformation is associated with diverse molecular changes which include alterations in the expression and activity of membrane channels and transporters (Herve 2015). Although still in its infancy, there is growing interest in understanding these changes within particular cancer types and exploring how modulation of these channels might lead to a novel treatment modality. The clinical relevance of TRP channel gene expression has recently been investigated (Park et al. 2016). Here, we focus on the emerging data related to TRPC4 and TRPC5.

### Relationship to vascular endothelial growth factor (VEGF) signalling and angiogenesis

Tumour angiogenesis is a hallmark of cancer and, as such, represents a promising therapeutic target. Currently used VEGF pathway-targeted drugs, such as the VEGF receptor tyrosine kinase inhibitors, are, however, not effective in all cancers. Increased understanding of VEGF signalling and the mechanisms underlying tumour vascularisation is required in order to realise the full potential of this strategy (Vasudev and Reynolds 2014).

Several studies suggest roles of TRPC channels in angiogenesis. Knockdown of TRPC4 and TRPC5 inhibited tube formation in an endothelial cell line (Antigny et al. 2012). Furthermore, a role for TRPC4 in the development of retinal neovascularisation has been suggested. TRPC4 was up-regulated in hypoxia, intravitreal injection of TRPC4 short interfering RNA reduced VEGF-induced retinal neovascularisation in oxygen-induced retinopathy, and TRPC4 short interfering RNA suppressed proliferation and Matrigel-based tube formation of human dermal microvascular endothelial cells (Song et al. 2015). Consistent with these observations and the involvement of heteromeric channels, TRPC1 gene-disrupted zebrafish showed disrupted VEGF-dependent angiogenic sprouting (Yu et al. 2010). TRPC1 knockdown also suppressed migration and proliferation in endothelial progenitor cells (Kuang et al. 2012). However, the TRPC1 inhibitor antibody had relatively little effect against VEGF-evoked Ca^2+^ entry of some types of endothelial cell, where Orai1 channels instead played roles (Li et al. 2011). Moreover, down-regulation of TRPC4 has been suggested as a trigger for tumour angiogenesis in renal cell carcinoma via a mechanism involving reduced secretion of the angiogenesis inhibitor thrombospondin-1 (Veliceasa et al. 2007). The implications of these channels for tumour angiogenesis require further investigation, and there is a significant possibility of both negative and positive implications depending on the context and contributions of other Ca^2+^-permeable channels.

### Potential role of TRPC5 in chemotherapy resistance

A special role for TRPC5 has been suggested in the development of resistance to cancer chemotherapy. TRPC5 and the multi-drug resistance (MDR) transporter, p-glycoprotein, were found to be up-regulated in the MCF-7 breast cancer cell line following repeated exposure to adriamycin until development of drug resistance (Ma et al. 2012). Inhibition of TRPC5 suppressed the effect on p-glycoprotein expression, leading to the suggestion that up-regulation of p-glycoprotein is downstream of TRPC5 channel activity. The effect was observed not only in vitro but also when MCF-7 cells were used in xenograft studies in mice. A similar effect was observed with the drug paclitaxel.

Subsequent research by the same group found TRPC5 in extracellular vesicles of the MCF-7 cells and suggested that chemotherapy resistance might be transferred to other cancer cells through these vesicles and the introduction of TRPC5 into other cells (Ma et al. 2014). Histological analysis of breast cancer tissue from patients before and after chemotherapy provided support for the idea that a similar phenomenon occurs in tumours of patients. The investigators suggested that identification of tumour-specific TRPC5 in circulating extracellular vesicles might provide a window on the clinical outcome of chemotherapy. In a related study, ATP-binding cassette subfamily B member 1, a member of the MDR family of proteins, was found to be up-regulated because of TRPC5 channel activity in colorectal carcinoma cells (Wang et al. 2015).

### Activation of TRPC4/5 channels by (−)-englerin A (EA) and the relationship to renal cell carcinoma cells

**Fig. 1:**
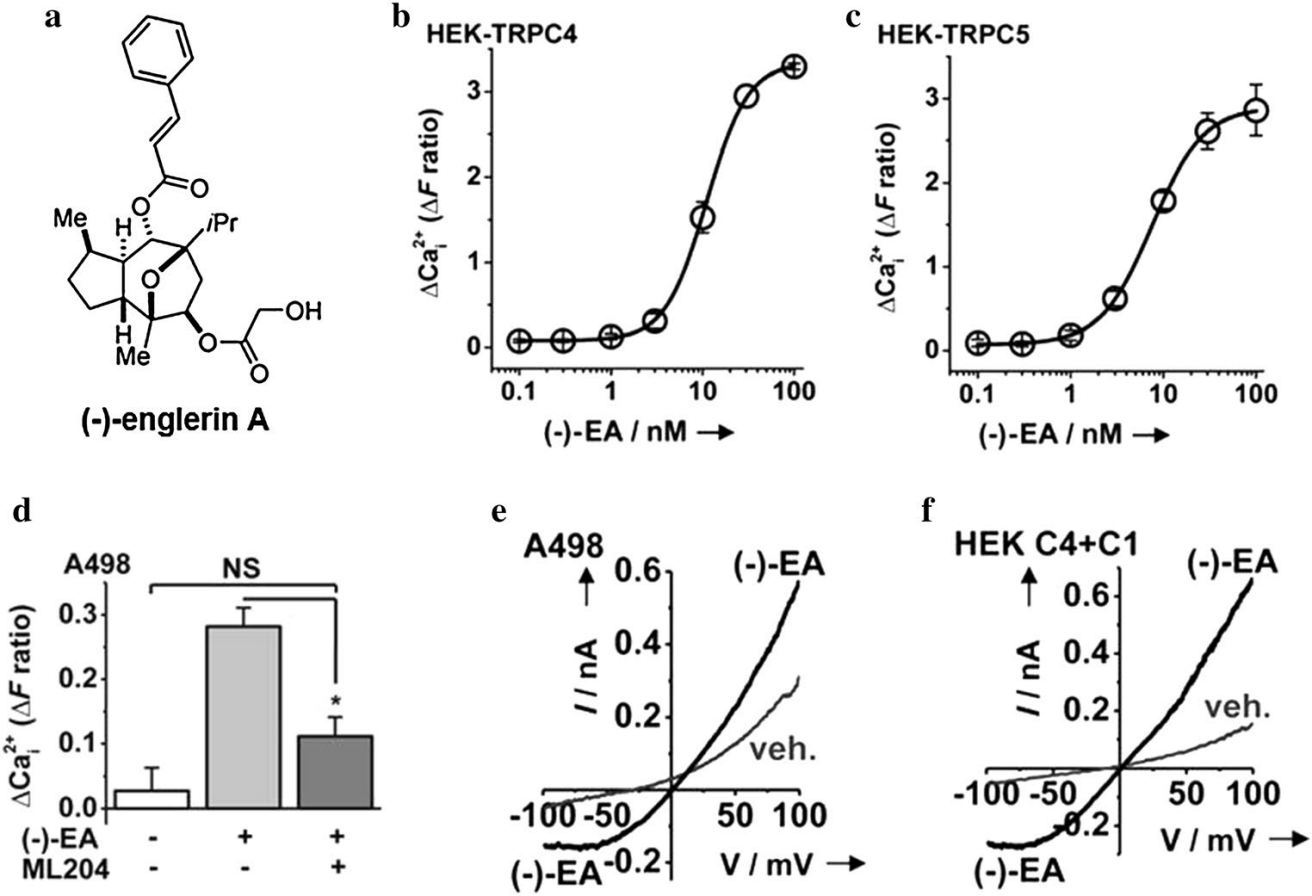
Discovery of (−)-englerin A (EA) as a novel potent and efficacious TRPC4/5 channel activator. **a** Chemical structure of EA (Akbulut et al. 2015). **b**–**d** Measurements of the free intracellular calcium ion (Ca^2+^) concentration shown as the change (Δ) in fura-2 fluorescence. **b** Concentration–response data for EA in HEK cells over-expressing TRPC4 (HEK-TRPC4) indicating the 50 % maximum effect (EC_50_) at 11.2 nM (Akbulut et al. 2015). **c** As for **b** except the cells were genetically modified HEK 293 cells induced to over-express TRPC5 (HEK-TRPC5). The fitted curve is a Hill equation indicating an EC_50_ of 7.59 nM (Akbulut et al. 2015). **d** Mean responses after 4 min exposure to vehicle, 1 µM EA, or 1 µM EA in the presence of 5 µM ML204 (Akbulut et al. 2015). **e** Whole-cell current–voltage relationship of membrane current from a single A498 cell during ramp changes in membrane voltage from −100 to +100 mV applied every 10 s. 100 nM EA or its vehicle were bath-applied (Akbulut et al. 2015). **f** As (**e**) except with genetically modified HEK 293 cells induced to over-express TRPC4 and transiently express TRPC1 (HEK C4 + C1) (Akbulut et al. 2015)

Bioactive natural products have proved to be valuable starting points for drug discovery, and screening efforts are continuously on-going to find previously unrecognised natural products with potent and potentially useful effects. The diverse genus *Phyllanthus* is a known source of biologically active compounds. One of them, *Phyllanthus engleri*, grows in South East Africa, where the fruit is eaten, tea is made from the leaves as a remedy for common ailments, and burning of the roots generates a toxic smoke. About 7 years ago, EA (Fig. 1a) was isolated from this plant and included in a screen against the NCI-60 cancer cell line panel (Ratnayake et al. 2009). It was found to have a rapid cytotoxic effect on certain types of cancer cell line at nanomolar concentrations. In particular, renal cell carcinoma cell lines were affected as well as a triple-negative breast cancer cell line (Ratnayake et al. 2009). Subsequent studies confirmed the cytotoxic effect on these cell lines (see below). Because of these effects, there were then major medicinal chemistry efforts to devise methods for efficient synthesis of EA (Radtke et al. 2011) and to find its protein target or targets (Sourbier et al. 2013; Akbulut et al. 2015; Carson et al. 2015).

Sourbier et al. suggested that EA activates protein kinase C θ to starve cells of glucose (Sourbier et al. 2013). However, Akbulut et al. found that this protein kinase C was not expressed in one of the most EA-sensitive renal cancer cell lines (Akbulut et al. 2015). Initial affinity-based chemical proteomics studies yielded no specific target, an explanation for which was considered to be that the target is a low-abundance membrane protein such as a G protein-coupled receptor or ion channel. This led to identification of TRPC4 and TRPC5 channels as targets of EA (Akbulut et al. 2015), and these targets were subsequently confirmed independently by another group (Carson et al. 2015).

EA turned out to be a remarkably efficacious, potent, specific and stereo-selective activator of TRPC4 and TRPC5 channels and TRPC1/TRPC4 and TRPC1/TRPC5 heteromeric channels (Akbulut et al. 2015). As little as 3 nM EA was enough to activate Ca^2+^ entry through TRPC4 channels or TRPC5 channels over-expressed in human embryonic kidney (HEK) 293 cells. The EC_50_ values for TRPC4 and TRPC5 were 11.2 and 7.6 nM (Fig. 1b, c). EA activated the channels from the outer face of excised membrane patches in the absence of co-factors, consistent with a direct action on the channels. In the A498 renal cell carcinoma cell line, EA evoked Ca^2+^ entry through endogenous channels with an EC_50_ of 9.5 nM, and this response was suppressed by ML204, the small-molecule inhibitor of TRPC4 channels (Fig. 1d). Whole-cell patch-clamp recordings suggested that the channels activated were most probably heteromers of TRPC1 and TRPC4, because the reversal potential and shape of the current–voltage relationship were similar to those of over-expressed TRPC1/TRPC4 channels (Fig. 1e, f). Importantly, the cytotoxic effect of EA could be reconstituted in otherwise resistant cells by over-expressing TRPC4 or TRPC5 and suppressed in cancer cell lines by knockdown of TRPC4 (Akbulut et al. 2015; Carson et al. 2015).

Although the initial study suggested that EA at 5 mg kg^−1^ lacked toxicity in vivo in mice and was effective against xenograft tumours (Sourbier et al. 2013), we found toxicity at 5 mg kg^−1^ (but not 2 mg kg^−1^) (unpublished data) and Carson et al. expressed concern about toxicity in rodents depending on the route of administration and dose (Carson et al. 2015). One challenge has been the formulation for in vivo studies, but a more serious problem has been the metabolic instability of EA, making it difficult to establish a meaningful dosing regimen (Carson et al. 2015). After administration of EA to rats at 5 mg kg^−1^, EA was detected in the blood at no more than 12 nM (Carson et al. 2015). This concentration is quite low, but it would be expected to activate endogenous TRPC1/TRPC4 channels based on in vitro data for A498 cells (Akbulut et al. 2015). We await studies on more metabolically stable EA derivatives which are active at the channels and information on whether the toxicity is mediated by TRPC4- or TRPC5-containing channels. At this stage, we can say that, in principle, it turns out to be possible to achieve rapid cytotoxicity through a potent and highly efficacious activator of TRPC4/TRPC5-containing channels and that such an agent might have potential as a starting point for a novel anti-cancer drug. There is, nevertheless, more research needed if the in vivo challenges of EA are to be overcome.

The effect of EA was initially emphasised in renal cell carcinoma cells, but it is also active in other but not all cancer cell lines, including cells derived from patients with triple-negative breast cancer and Ewing’s sarcoma (Ratnayake et al. 2009; Carson et al. 2015; Caropreso et al. 2016).

### Inhibition of TRPC4-containing channels in cancer cell lines and the EA paradox

In non-small cell lung cancer, the expression of TRPC1 and TRPC4 was suggested to correlate with tumour grade (Jiang et al. 2013) and treatment with short interfering RNA targeted to TRPC1 and TRPC4 or inhibitor antibodies suppressed proliferation of an ovarian carcinoma cell line (Zeng et al. 2013). Conversely, over-expressing TRPC1 or TRPC4 increased proliferation (Zeng et al. 2013). These studies support the idea that TRPC4-containing channels are functionally significant in certain types of cancer cell line. They also reinforce the apparent paradox of the EA findings, where activation of the channels leads to rapid cell death amongst certain cancer cell lines. One explanation could be that it is simply a matter of the bell-shaped relationship between intracellular Ca^2+^ concentration and cell function, whereby modest elevations of Ca^2+^ are beneficial for cells, encouraging proliferation and migration, whereas high elevations, for example about 1 µM, are essentially toxic for cells, encouraging apoptosis and necrosis (Orrenius et al. 2003); that is, inhibition of basal activity of the channels might inhibit at least part of the unwanted proliferation and migration in cancer cells, whereas strong activation by a substance like EA might cause rapid cytotoxicity.

## Conclusions

**Fig. 2:**
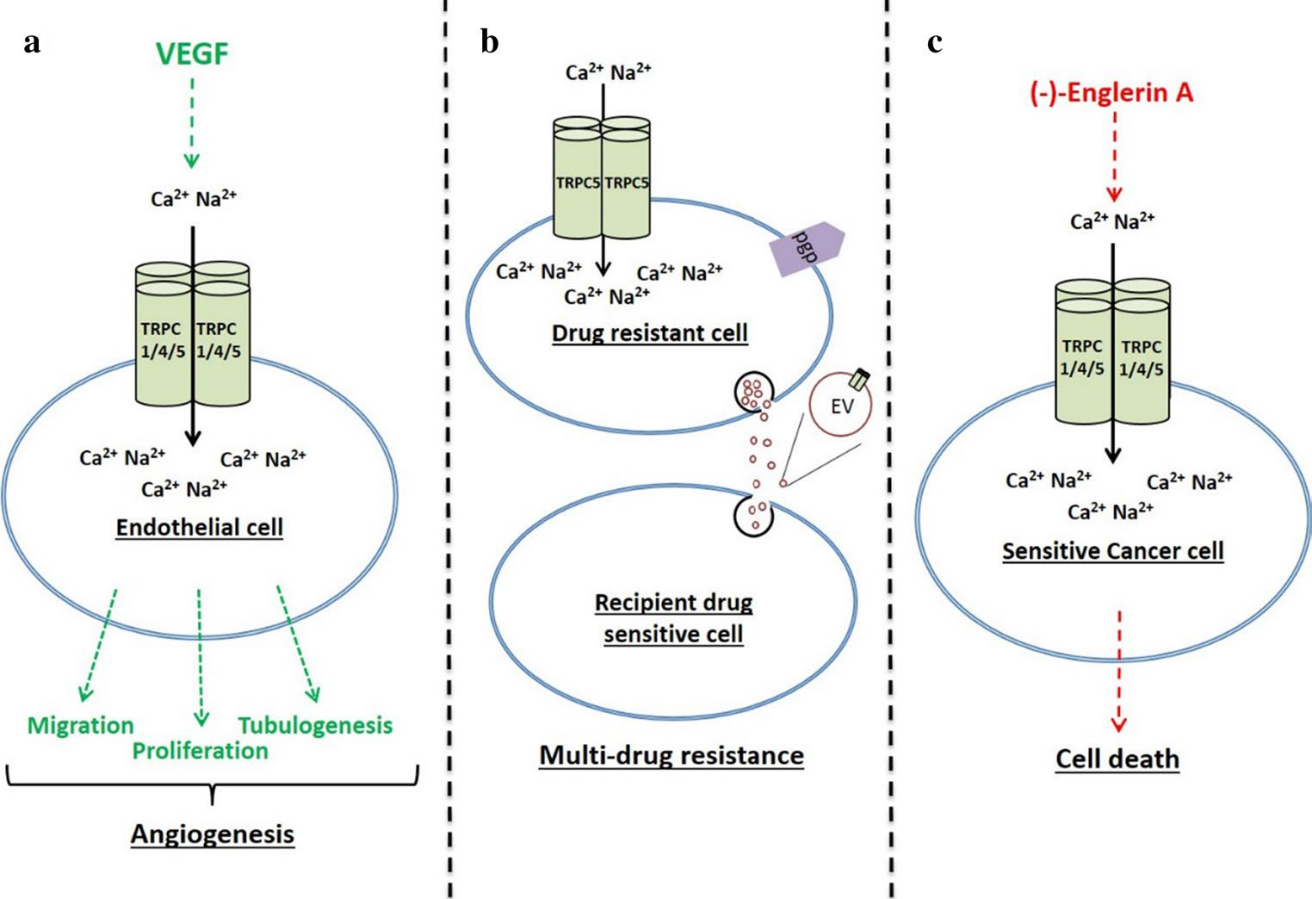
Simplified overview of TRPC4/5 channels as potential therapeutic targets in cancer. **a** VEGF-activated TRPC1/4/5 channels allow non-selective cation entry. A modest rise in intracellular Ca^2+^ can lead to an increase in migration, proliferation and tubulogenesis of endothelial cells, leading to angiogenesis. **b** TRPC5 drives chemoresistance in breast cancer cells as it leads to up-regulation of p-glycoprotein (pgp) which acts to pump drugs from the cell. TRPC5 is expressed in extracellular vesicles (EVs), and a critical role of TRPC5-containing EVs is in the transfer of drug resistance to non-chemoresistant recipient cells.** c** (−)-Englerin A is a selective TRPC1/4/5 channel activator which causes influx of Ca^2+^ and Na^+^ into certain types of cancer cell, which then causes cell death

There are still few studies of TRPC4 and TRPC5 and TRPC4- and TRPC5-containing channels in cancer cell lines and even fewer on human cancer itself (Table 1). Therefore, any conclusions can only be preliminary. The studies reported so far do, nevertheless, suggest that there might be benefits for some cancer patients in taking a medication that inhibits or, paradoxically, activates these channels. Resistance to chemotherapy, cancer cell proliferation, cancer cell migration and tumour vascularisation might be suppressed, as well as the potential for rapid induction of selective cytotoxicity in certain types of cancer cell, such as those of renal cell carcinoma (Fig. 2). It should be emphasised, however, that most of the data supporting such suggestions have arisen from studies of cancer cell lines, which may often differ substantially from the cancer cells populating the tumours of patients. Moreover, even if suitable small-molecule modulators of the channels can be identified, it needs to be recognised that TRPC4- and TRPC5-containing channels are just one mechanism by which a cancer cell might regulate the intracellular Ca^2+^ required for Ca^2+^-dependent cell processes; that is, it is quite conceivable that modulation of the channels could relatively easily be circumvented by an ever-adapting cancer cell.

**Table 1:** TRPC1/4/5 in cancer

	TRPC4	TRPC5	TRPC1
Migration/proliferation of cancer cells	Ovarian carcinoma cell line proliferation (Zeng et al. 2013)		Ovarian carcinoma cell line proliferation (Zeng et al. 2013)
Angiogenesis	Tube formation of endothelial cell lines in a Matrigel assay (Antigny et al. 2012)	Tube formation of endothelial cell lines in a Matrigel assay (Antigny et al. 2012)	Angiogenic sprouting in zebrafish (Yu et al. 2010)
	Tube formation and proliferation of human dermal microvascular endothelial cells (Song et al. 2015)		Vascular repair; endothelial progenitor cell migration and proliferation (Kuang et al. 2012)
	Retinal neovascularisation induced by VEGF (Song et al. 2015)		
	TRPC4 down-regulation in renal cell carcinoma enables the angiogenic switch (Veliceasa et al. 2007)		
Multi-drug resistance		Overexpressed in drug-resistant breast cancer cells and is involved in adriamycin resistance (Ma et al. 2012)	Resistance of multi-drug-resistant breast cancer due to involvement in regulation of ABCC3 expression (Stewart et al. 2015)
		Role for TRPC5 in trafficking of extracellular vesicles from resistant to wild-type cells causing chemoresistance (Ma et al. 2014)	
		ABCB1 induction and drug resistance in colorectal carcinoma (Wang et al. 2015)	
Cell death	TRPC4 channels are a target of (−)-englerin A (Akbulut et al. 2015)	TRPC5 channels are a target of (−)-englerin A (Akbulut et al. 2015)	TRPC1/4/5 channels are a target of (−)-englerin A (Akbulut et al. 2015)
	(−)-Englerin A-activated TRPC4 channels are involved in its mechanism of killing renal cell carcinoma cells (Carson et al. 2015)		

The roles of TRPC4/5/1 in cancer cell migration and proliferation, angiogenesis, multi-drug resistance and cell death

Despite this recommendation for caution, we conclude that TRPC4 and TRPC5 represent potentially attractive targets for cancer therapeutics. Their diverse role in many of the aspects that drive the metastatic process warrants continued research into their context-dependent function. It is already apparent that inhibitors of the channels would be unlikely to cause significant adverse effects. Instead, it is conceivable that they may be associated with other benefits to patients with advanced malignancy, including suppression of innate fear, pain and pathological cardiac remodelling, where inhibition of these channels may be beneficial (Camacho Londono et al. 2015; Phelan et al. 2013; Zheng and Phelan 2014; Riccio et al. 2014; Wei et al. 2015; Westlund et al. 2014; Bon and Beech 2013).

## Abbreviations

TRPC: Transient receptor potential canonical
TRPV: Transient receptor potential vanilloid
TRPM: Transient receptor potential melastatin
TRPA: Transient receptor potential ankyrin
TRPP: Transient receptor potential polycystin
TRPML: Transient receptor potential mucolipin
2-APB: 2-Aminoethoxydiphenyl borate
VEGF: Vascular endothelial growth factor
EA: (−)-Englerin A
HEK: Human embryonic kidney
MDR: Multi-drug resistance
EVs: Extracellular vesicles
